# An Empirical Study of the Impact of the Air Transportation Industry Energy Conservation and Emission Reduction Projects on the Local Economy in China

**DOI:** 10.3390/ijerph15040812

**Published:** 2018-04-19

**Authors:** Yuxiu Chen, Jian Yu, Li Li, Linlin Li, Long Li, Jie Zhou, Sang-Bing Tsai, Quan Chen

**Affiliations:** 1 College of Civil Aviation, Nanjing University of Aeronautics and Astronautics, Nanjing 211106, China; yxchen@cauc.edu.cn; 2 Research Center for Environment and Sustainable Development of China Civil Aviation, Civil Aviation University of China, Tianjin 300300, China; 15522526057@163.com (Lin.L.); lilongs010@163.com (Lon.L.); 3 Wuxi Audit Division, Bank of Ningbo, Wuxi 214043, China; 151758@nbcb.cn; 4 College of Tourism and Service Management, Nankai University, Tianjin 300071, China; 5 Zhongshan Institute, University of Electronic Science and Technology of China, Guangdong 528400, China; zschenquan@gmail.com

**Keywords:** energy conservation and emission reduction, benefits, air transport, input-output method, green environment, green operation, sustainable development

## Abstract

Green development has been of particular interest to a range of industries worldwide, one of which being the air transportation industry (ATI). The energy conservation and emission reduction (ECER) projects of the ATI have a huge impact on the local economy. In this study, the input-output method was used to analyze the indirect economic impact of the implementation of the ECER projects of the ATI on the local economy of the Beijing-Tianjin-Hebei (BTH) region. We examined the direct benefits, backward spread effects, forward spread effects, and consumption multiplier effects. The final results showed that the comprehensive economic income from 2011–2013 in the BTH region reached RMB 4.74 billion. The results revealed that the ECER projects commissioned by the ATI were worth investing from both the economic and social benefits perspectives. To increase the green development effects and promote the sustainable development of the ATI, the special funds provided by the Civil Aviation Administration of China should be invested intensively in basic green technology research and setting green regulating and governance rules.

## 1. Introduction

A low carbon economy has been evolving to become an emerging and important competitive method worldwide. The International Civil Aviation Organization (ICAO) and European Union (EU), as well as other international or regional organizations have proposed much higher targets for Energy Conservation and Emission Reduction (ECER) in air transport. In these circumstances, green and low carbon development has already been a necessary consequence, and one of the most important developing approaches in the Chinese air transportation industry (ATI). Furthermore, the Civil Aviation Administration of China (CAAC) has also proposed an average goal of an annual 4% reduction of fuel consumption and carbon dioxide release per ton-kilometer during the Chinese 13th Five-Year Term, when compared with the 12th Five-Year Term [1]. In order to fulfill these objectives, the application of innovative technologies and renewable energies as well as instrument upgrades and innovative administration policies are the inevitable means. However, global experiences have proved that the costs of implementing these measures are extremely high, not only in the ATI. Therefore, such costs and expenses of emission control have obviously been given serious attention by researchers and practitioners.

With the Global 2100 framework (which was used to assess the overall economic impact of energy costs increase), Manne and Richels evaluated the influence of price of premium crude oil on conventionally measured GDP [2]. They found that China was most strongly affected by the higher energy costs regulated in the international carbon reduction agreements. In order to undertake more precise calculations with the Computable General Equilibrium model in the future, this research also carried out some useful attempts including the future variation trends of oil price. Based on their findings, Manne and Richels proposed the implementation of a carbon tax to reduce the consumption of carbon-intensive fuel. To assess the control costs that the governments and society should pay to prevent climate change, Maddison constructed a dynamic non-linear program with the objective of minimizing the control costs [3]. Emissions reductions and sink enhancement were the two key control variables in the model, and Maddison put forward several proposals to hold back climate change based on these findings, for example, afforestation, decreasing emissions, imposing carbon tax, and removing fossil fuel producer allowances. Fan et al. also used the objective programming approach to estimate the costs of CO_2_ emission control in China and indicated that the costs included the investment in emission reduction and also the losses due to limiting the development of high-emission sectors [4]. Similarly, several other researchers have assessed the cost of reducing CO_2_ emissions in other regions by the same means, for example, the study of Hsu and Chou in Taiwan [5]. The cost model is another commonly used technique to appraise the expense of emission control. With the help of a production cost model based on the large panel data of four high-consuming energy industries, Morgenstern et al. compared the expenditure for the environmental protection of plants between the reported expense and the actual burden [6]. However, they could not find consistent results as to whether the reported expenses of the individual plants were overstated or understated in terms of the actual costs. Such a finding was just another indication of the requirement to assess the emission control cost/income more precisely. Comparing the whole economic costs of renewable sources of electricity energy with that of coal-fired power plants, Crane et al. found that the marginal cost of renewables grew rapidly after Greenhouse Gas emissions were reduced by renewables by up to 100 million metric tons and the total annual cost of 25% of the Renewable Portfolio Standards in U.S. would be USD $35 billion [7]. The results also indicated that the substitution of renewables for coal-fired electricity would prove economical only if the renewables technologies were favorable, and this also emphasized the significance of the strategies to motivate the development of renewable technologies (capital expenditure expansion of research and development, for example). Undoubtedly, the inputs and costs of green and low carbon development cost a large amount in current technical circumstances all over the world. Unfortunately, the direct economic income of the new technology will be much lower, even if the effects of ECER were still not stable and obvious in practice. Using the conservation supply curve and evaluating the technologies cost of conserved energy (CCE), which includes the investment, operating costs, maintenance costs, and subtracted the cost of saved energy, Yuan and Lei found that almost half of the technologies used in the iron and steel sector in China were not cost-effective, and their CCEs were over zero [8].The results of similar researches in the ATI reach more or less the same outcomes. By calculating the Data Envelopment Analysis value with the Super Efficiency Data Envelopment Analysis model, Chen and Yu found that a large proportion of technologies and improvement measures in ECER projects in the ATI of Northern China were not relatively effective [9]. The non-economic effectiveness or non-economic efficiency in the short-term suggested the local governments and industry policymakers needed to be more prudent when making decisions as to whether or not to promote these neo-technologies.

In the ATI of China, aircraft fuel consumption represents 94% of the entire energy expense [10]. The cost of fuel accounts for more than 40% of the total operating costs of the Chinese airline industry [11]. If it is cost effective in accounting, there will be a much larger motive to invest in and promote the energy saving projects. As a result, policy makers and especially the airline managers will pay more attention to aircraft fuel conservation measures such as bio-oil techniques, or the optimal means to cut fuel costs, which was the only deducted income element in Li and Zhu’s CCE model [8]. Many studies have also been devoted to assessing the fuel consumption efficiency of aircrafts [12,13,14,15]. The Civil Aviation Administration of China (CAAC) also cares more about the fuel or energy efficiency. During the 12th Five-Year Plan period, the CAAC arranged more than RMB 2 billion to support the airlines, airports, and other institutions to improve energy efficiency and decrease the exhaust gas emissions. However, should the short-term economic benefits of implementing energy saving technologies only include the cost of saved energy? Is such a large amount of funding economically effective? How much in subsidies should the government air transportation departments in China provide when they make investment decisions? The precise and comprehensive understanding and calculation of the economic income of ECER projects is very important when attempting to answer these questions. However, there are few studies in such fields in China. The purpose of this paper is to clearly state and evaluate the comprehensive economic income generated by the investments into ECER by the ATI in China.

## 2. Literature Review

The economic benefits can be divided into the direct income and the indirect income according to the benefitted range, into the macro income and the micro income according to the benefitted size, and into the short-term income and the long-term income according to the benefitted length of time [16]. To describe the implementation process of the benefits of energy conservation and the emission reduction projects of the ATI, the classification of the direct and indirect income was chosen in this paper.

The ATI is a type of producing sector and can also produce Gross Domestic Product (GDP) (in the input-output tables of China, the components of value added include the Compensation of Employees, the Net Taxes on Production, the Depreciation of Fixed Asset, and the Operating Surplus), which is called the direct income. When the airlines, airports or other utilities of the ATI develop and implement ECER projects, they may hire more employees and invest in some new instruments and equipment. Such projects may also alter the operating expenses (e.g., cutting the cost of fuel) and correspondingly influence income tax. All of these can be treated as the direct income generated by the ECER projects by the ATI.

The ATI is one component in an integrated traffic system and plays a more important role in the development of the local community economy [17,18]. Due to the close relationship with other producing sectors, the benefits created by the ATI are much larger than the income generated on its own. The changed income of the ATI produced by ECER projects will spread to other producing sectors with different correlative patterns. The corresponding influence on the income of other sectors can be called the indirect income of the ECER projects by the ATI. The R&D and implementation of ECER projects by the ATI require a good deal of input in new techniques and facilities and instruments as well as a large amount of various kinds of materials and alternative energy sources (e.g., electricity, natural gas, and biofuel). Therefore, the production of the related producing sectors of the ATI will expand and consequently enlarge their demands of the producing inputs. This chain reaction will diffuse to many other industries. Such phenomena can be called the backward spread effects (BSE) of ECER projects by the ATI. Similarly, the ATI is also an important producing unit of other industries and the continuous process of producing in the circulation domain. The implementation of ECER projects can not only decrease emissions, but also help the airlines or airports cut operating costs. Furthermore, it provides the whole industry with an excellent chance to develop new technologies and extend the business and scope of the transportation network. Positive results create advantages that help other producing sectors to expand their production scale, with the capability to transport more passengers, commodities, and materials. According to the theory of the balance of the national economy, the production expansion of other producing sectors also requires more commodities and materials from their intermediate input supplying sectors. Likewise, the chain reaction will also pervade the whole national economic system. These kinds of benefits are the forward spread effects (FSE) of ECER projects by the ATI. The BSE and the FSE of ECER projects of ATI will also give rise to increased employee salaries due to the enlarged production of the entire producing sector. The increased income of the residents will then generate a new round of consumption and stimulate the production once again. These cyclical effects can be called the consumption multiplier effects (CME). All the BSE, FSE, and CME are the indirect benefits of ECER projects by the ATI.

The value added approach [19], the regression model [20,21], and the simultaneous equations [22] are the most commonly used methods when estimating the influence of air traffic on the local economy. The input-output method (I-O) is another approach that is usually used in many fields to evaluate the macroeconomy such as the economic benefits, the growth ability, and the evolutionary features of some industries [23,24,25,26,27]. For understanding the strong interrelationship with other producing sectors, I-O analysis is suitable for research into the economic effects of transport issues. Hence, the International Civil Aviation Organization (ICAO), which is a specialized UN agency, established by member states in 1944 to manage the administration and governance of the Convention on International Civil Aviation (Chicago Convention), used the I-O method to evaluate the contribution of air transport to the local economy and its findings indicated that every $100 of output produced and every 100 jobs generated by air transport will trigger additional demand of some $325 and 610 jobs in other industries [28]. The Federal Aviation Administration of the U.S. (FAA) also adopted the I-O model to appraise the induced economic impacts of the expenditure by the air transportation industry and their results showed that commercial aviation contributed $807.1 billion or 5.1% to the U.S. GDP in 2012 [29].

Given its strong applicability, the analytical framework and I-O analysis models were adopted in this paper. This study proceeds as follows: Section 3 describes the methodology, Section 4 describes the data and discusses the findings of the study, and Section 5 draws the conclusions and provides some discussion in this area.

## 3. Methodology

The I-O method was proposed by Leontief in 1936. In the national economic system, the production of each sector needs the outputs from other sectors as the necessary operating resources [30,31,32,33,34,35,36]. At the same time, the outputs of each sector are also important operating inputs for other production sectors in the national economic system. The I-O method adopts a chessboard input-output table to reflect the movement processes of the production among various producing sectors from the consumption and distribution aspects [37,38,39,40,41,42,43]. Table 1 shows the basic Chinese I-O table form. The I-O table can be mainly split into three parts: The I Quadrant, the II Quadrant, and the III Quadrant. In the trans-verse direction, the I Quadrant shows the service or production provided by one sector to the other sectors’ producing process. In the vertical direction, the I Quadrant reveals the service or production produced by other sectors that one sector consumes in their producing process. Hence, the I Quadrant is an important information source of the intermediate inputs, the intermediate use, and the I-O relationships between various producing sectors. The II Quadrant reveals the final use of the gross value of production after distribution and redistribution, and contains the final consumption expenditure and the capital formation. The intermediate use in the I Quadrant and the final use in the II Quadrant can provide the allocation and utilization of the total goods and service produced by the whole economic system [44,45,46,47,48,49]. The III Quadrant shows the formation and composition of the Value Added, which includes the compensation of employees, the net taxes on production, the depreciation of fixed assets, and the operating surplus. Such a constitution can help to calculate the impact of the variation of any industry, including the ATI, to the total macro-economy. The intermediate inputs in the I Quadrant and the value added in the III Quadrant are equal to the total economic inputs and reflect the value composition of the productions or services of each producing sector.

The I-O table has two patterns of manifestation: the physical I-O table and the value I-O table. The value I-O table is widely used because the physical I-O table can only reveal the practical quantitative relationship. The value dependence relationship revealed by the value I-O table can be described through a system of linear equations. Furthermore, there are always several coefficients to be introduced to the system of linear equations to express the techno-economic relationships among various producing sectors and help measure the value quantitative relationships between the general products and the intermediate products or/and the final products. These coefficients were adopted in this study to assess the direct income, BSE, FSE, and CME of the ECER by the ATI to the macroeconomy [17,18,30].

### 3.1. Direct Income Model

Let $d_{p}$ be the direct income of any producing sector *p*. The direct income model is:

(1) $$d_{p}={\bar{Z}}^{T}\Delta X$$

where *ΔX* is the output appreciation vector of each producing sector and ${\bar{Z}}^{T}$ is the value-added coefficient vector. The changed output of the air transportation industry caused by the ECER projects, $\Delta X_{t}$, is paid much more attention in this article. Since only air transport sector *t* was measured, the other sectors’ output was set to 0, and *ΔX* is described as ${\left(0,\ldots ,\Delta X_{t},\ldots ,0\right)}^{T}$. The value-added coefficient of sector *p*, $\bar{Z_{p}}$, represents the value of GDP generated by a single output of sector *k* and can be described as $z_{p}/X_{p}$. *z_p_* is the added value generated by sector *p*, including the compensation of laborers, net taxes on production, depreciation of fixed assets, and operating surplus, just the components of each sector in the III Quadrant. *X_p_* is the output of sector *p*.

### 3.2. BSE Model

Let *b_p_* be the BSE of any producing sector *k*. The BSE model is:

(2) $$b_{p}={\bar{Z}}^{T}B\Delta X$$

where *B* is the complete consumption coefficient matrix. The complete consumption coefficient is the total consumption—the direct consumption and indirect consumption—of other sectors when producing a single output of sector *p*. *B* can be described as $（I-A{）}^{-1}-I$, where *A* is the direct consumption coefficient matrix, and *I* is the identity matrix. The direct consumption coefficient *a_ip_* indicates the amount or value of product *i* consumed when manufacturing one unit of product *p*. ${\bar{Z}}^{T}B$ is called the BSE multiplier.

### 3.3. FSE Model

Let *f_p_* be the FSE of any producing sector *p*. The BSE model is:

(3) $$f_{p}=\Delta X^{T}\times Q\times {\bar{Z}}^{T}$$

where *Q* is the complete partition coefficient matrix. The complete partition coefficient shows the share of certain sectors’ production allocated to other sectors through direct and indirect patterns. *Q* can be calculated as ${\left(I-H\right)}^{-1}-I$*,* where *H* is the direct partition coefficient matrix. The direct partition coefficient *h_pi_* indicates the amount or value of one unit product *p* distributed to manufacture product *i*. $Q.{\bar{Z}}^{T}$ is called the FSE multiplier.

### 3.4. CME Model

Keynes proposed the Multiplier Theory based on the Consumption Propensity Principle and derived the quantitative relationship between the gross national income and the investment. That is, the impact of the investment variation on the gross national income is much larger than the investment variation itself. The ratio between the variation of the gross national income and the investment is called the invest multiplier. The increment of the gross national income generated by the augment of investment also includes the incremental indirect consumption due to such activities. Hence, the invest multiplier is connected with the consumption propensity. According to Keynes’s multiplier principle, the invest multiplier can be described as 1/(1−*c*), where *c* is the marginal consumption propensity [31]. The function of *c* is $\frac{\sum C}{\sum G}$, where ∑C is the total consumption of the whole society and ∑G is the total GDP [32]. Let $c_{k}$ be the CME of any producing sector. Then, the consumption augment generated by the direct income, BSE, and FSE is:

(4) $$c_{k}=\left(d_{k}+b_{k}\;+\;f_{k}\right)\frac{c}{1-c}$$

$\frac{c}{1-c}$ is called the consumption multiplier. Summing up all the direct income, BSE, FSE, and CME, the economic impact of ECER projects of by the ATI (EIEA) on the total society can be obtained, that is:

(5) $$EIEA=\left({\bar{Z}}^{T}\Delta X+{\bar{Z}}^{T}B\Delta X+\Delta X^{T}\times Q\times \;{\bar{Z}}^{T}\right)\times \left(1+\frac{c}{1-c}\right)$$

## 4. Data and Results

CAAC has arranged special funds annually since 2011 to subsidize and support the entities of ATI to research or/and implement ECER projects. These ECER projects can be categorized into seven groups: energy-saving technology improvement, energy saving by management measures, energy conservation products and green energy application, procurement or/and modification of aviation ground vehicle powered by green energy, air routes optimizing, disposal of airport sewage and waste water and procurement of reclaimed water facilities, and energy statistics and monitoring (There is another category named fundamental and strategic research projects, which generally funds the policies and strategies analysis. Due to the difficulties of measuring the direct income of such research items, this category was not included in our study). The units involved include airlines, airports, air controlling departments, supporting organizations, and other utilities. In 2014, CAAC inspected the execution of ECER in the seven regions (For the managing convenience and efficiency, CAAC established seven branches distributed in seven regions in China, named North China, North East China, North West China, Eastern China, Middle-South China, South West China and Xinjiang) all over China. The Beijing-Tianjin-Hebei region (BTH) is located in North China and has attracted the special attention of the central government of China as an entirety in recent years. The synergetic development of BTH was also highlighted in the report of the 19th CPC National Congress in November 2017. CAAC has also issued The Opinions on Promoting the Coordinated Development of Air Transportation of BTH. We attended the BTH inspecting team and for the data availability, BTH was used as a case in our study to describe the calculating process.

There were 124 projects implemented and funded by CAAC in BTH from 2011 to 2013, and their detailed status is listed in Table 2. 

Table 2 shows that the investment on the ECER projects of airlines occupied the major share of the total funds annually and the three-year average from 2011–2013 was 79.52%. This investment propensity just matched up the ECER features of the ATI, which was that the fuel burned and corresponding exhaust gas discharged by the airlines were the controlling emphasis in the ATI. It also indicated that the investment from the CAAC and the operating entities grew year by year. This suggests that the inputs and the attention on the ECER issues of the ATI have increased over time.

The operating surplus can also be alluded to as the operating results, which are the comparison results between the income and various daily expenditures [33,34]. The ECER projects by the ATI cannot create additional revenue directly. Their main objectives are to increase energy efficiency and save energy consumption. Hence, the income of the projects here are the energy costs saved by the ECER projects of the ATI, e.g., fuel consumption retrenching due to the use of winglets (winglets are devices installed on the wing tip of the aircraft; winglets can help to reduce the induced drag of wings and thus contribute to decrease the fuel consumption) projects. The daily operating costs of the ECER of the ATI not only include the daily instruments maintenance cost, but also the alternative additional energy expenses such as the incremental electric power expenditure because of the ground power unit (GPU) equipment substituted for auxiliary power unit (APU) equipment. Hence, the operating surplus in our study was calculated as the difference between the income and daily operating costs of each project. The main alternation of net taxes on production in the I-O table was due to the implementation of ECER projects by the ATI is the variation of business income tax [35]. According to the data from the Civil Aviation Statistics Yearbook prepared by CAAC, the percentage of income tax to the operating costs of the ATI from 2011 to 2013 was 1.56%, 1.29% and 1.16%, respectively. Additionally, the net taxes on production in our study were the production of the operating surplus and the percentage of income tax to the operating costs. The total capital invested on the ECER projects of the ATI mainly constituted the value of the different kinds of fixed assets such as the Winglets on the aircraft. We assessed the depreciation of these fixed assets according to the assets life time expected by each projects implementation institution with the direct depreciation method. The performance of the ECER projects by the ATI did not need to hire extra employees. Therefore, the compensation of employees was negligible. Then, the changed output of the air transportation industry caused by the ECER projects, $\Delta X_{t}$, can be obtained as the summation of the value of the four items above. Table 3 shows the results of the $\Delta X_{t}$ of annual new projects. It can be seen in Table 3 that the value of the annual additional $\Delta X_{t}$ in 2013 was 1.56 times and 6.18 times as much as the value in 2012 and 2011, respectively. This indicated that the economic outputs of the new ECER projects by the ATI increased as the inputs and the attention on the ECER issues of the ATI were improved.

The provincial input-output table has been formally drawn up by the provincial bureau of statistics of various local provincial government of China every five years since 1987. Currently, there are two formats of I-O table, which are the 42-sector table and the 139-sector table. The 42-sector table was expanded to a 139-sector table after 2002 to reflect the input-output relationship in much more detail. The air transportation sector is displayed separately in the 139-sector table. The latest provincial I-O tables including 139 sectors were published in 2012. Due to the objective of this study, the provincial input-output tables of 139 sectors for 2012, published by the local Bureau of Statistics of Beijing, Tianjin, and Hebei, were adopted, which was the closest to 2011 and 2013. Based on the method and ideas put forward by Jiang [36], we aggregated the data of I-O tables of these three provinces to get the 139-sector I-O table of BTH for 2012. Then we used the 139-sector I-O table of BTH for 2012 to calculate the I-O coefficients.

With the models above described, we calculated the value-added coefficient, the complete consumption coefficient, and the complete partition coefficient of the BTH. The complete results are shown in Appendix A. Table 4 summarizes the top 10 results of the complete consumption coefficient and the complete partition coefficient of the BTH. It was indicated that besides the Air Transport sector itself, the Manufacture of Refined Petroleum Products, Processing of Nuclear Fuel sector and the Extraction of Crude Petroleum and Natural Gas sector were the two sectors that Air transport sector is most reliant upon in BTH. The Manufacture of Other Transport Equipment sector is another sector that provides lots of support to the Air Transport sector in BTH. Such consequences are in line with the actual situation of the ATI. From the overall perspective, the fuel expense and the instruments operating expenditures separately occupied 40.1% and 22.4% of the total operating costs of airlines in 2014 [50]. Table 4 also reveals that the sector of Public Management and Social Organization and the sector of Cargo Handling and Transport Agency were the two sectors that consumed the most ATI products and services in BTH, aside from the Air Transport sector itself. At the same time, the ATI provides strong backing to the development of the tertiary industry in BTH, much like the high-tech industry and the financial industry.

Using the data of the annual statistical communiqué of the local economic and social development of Beijing, Tianjin and Hebei, the marginal consumption propensity and the consumption multiplier are calculated to be as showed in Table 5.

Next, we obtained the EIEA results of the BTH from 2011 to 2013. Table 6 shows the values in detail. This indicated that the EIEA was worth much more than the capital invested on the ECER projects of the ATI. The total EIEA of these three years was ¥3.52 billion and the comprehensive economic income of these three years, which is the sum of the EIEA and the accumulated operating surplus, reached ¥4.74 billion and was 7.56 times as much as the total funds and capital invested. The results are clear evidence that the ECER projects of the ATI not only generate emissions reduction and promote the green development of the whole industry, but contributed much more to the national economy due to the operating expense saved due to the drop in the energy and fuel consumption.

## 5. Discussion

It can be seen that in order to boost the yield of ECER projects, improving the direct operating surplus will be the key point. That is, strengthening the energy-saving potential and then cutting the operating energy consumption expenses of the projects’ executing units. It is precisely due to such effects that airlines have a greater interest in executing projects such as the modification of winglets or aeromotors to cut down on their fuel costs. The total investment of BTH from 2011 to 2013 on such equipment reconstruction items was ¥3.72 billion, which occupied 59.24% of the overall capital and funds. It should also be noted that the funds provided by CAAC on these types of instrument modification projects totaled ¥1.14 billion, which was 60.75% of the general CAAC finance on ECER projects in the BTH region. However, the recent practice and global revolution tendency of ECER showed that the innovation of fundamental technology (e.g., R&D on biofuel and advanced materials) and green development rules, policies and strategies were exactly the most pivotal solutions to increase the prospective ECER efficiency, especially in China. Such kinds of studies and actions cost a huge amount of funds and human capital, aside from the very long preparation and research time. Furthermore, the direct economic benefits usually do not appear in the short-term or the net value is negative, sometimes the R&D of such projects even falls through to nothing. They contain more characteristics of public goods and non-profits. There is no business willing to invest in economically unviable projects. In the modern national governance system, it is the duty of the governmental institutions, like the CAAC, to perform the function of regulating, governance, and providing public services. It needs to let the market take the decisive role in resource allocation, that is, the airlines themselves still have great desire to invest in and execute projects that can produce valuable returns even without the support of the CAAC. Hence, we suggest that the special funds of the CAAC in the green development field are better spent to intensively subsidize the areas of basic green technology research, setting green standards and other crucial policies and strategies (e.g., constructing the market for trading carbon emissions).

## 6. Conclusions and Future Research

We measured the comprehensive economic contribution of the ECER projects by the ATI in BTH with the I-O method. The results showed that such capital and funds invested on these projects produced more direct and indirect income. Hence, the ECER projects are worth investing in from the perspectives of both the economic and social benefits. The overall gains assessed could be one of the judgement foundations for governmental departments like CAAC and other entities in the ATI when making investment decisions regarding ECER projects.

A uniform carbon emissions trade scheme (ETS) has not been established in China up until now. At present, there are only some experimental markets in several provinces. Therefore, the operating surplus in this study was only calculated as the difference between the energy costs saved and the daily operating costs of each project. Once a formal Chinese ETS has been set up and all market entities are participants, the operating surplus should extend to cover such income and loss. Meanwhile, the impacts of Chinese ETS to the green development of ATI are also worthy of a large amount of work.

## Figures and Tables

**Table 1:** Basic input-output table form.

Output		Intermediate Use				Final Use										Imports	Errors	Total (Gross) Output
Input		Sector 1	……	Sector 139	Total Intermediate Use	Total Final Consumption Expenditure					Gross Capital Formation			Exports	Total Final Use			
						Household Consumption Expenditure			Government Consumption Expenditure	Total	Gross Fixed Capital Formation	Changes in Inventories	Total					
						Rural Household	Urban Household	Total										
Intermediate Inputs	Sector 1	Ⅰ Quadrant				Ⅱ Quadrant												
	……																	
	Sector 139																	
	Total Intermediate Inputs																	
Value Added	Compensation of Employees	Ⅲ Quadrant				-												
	Net Taxes on Production																	
	Depreciation of Fixed Assets																	
	Operating Surplus																	
	Total Value Added																	
Total Inputs																		

**Table 2:** The overview of Energy Conservation and Emission Reduction projects in Beijing-Tianjin-Hebei region from 2011 to 2013.

Year	Airlines Projects Investment (Ten Thousand ¥)	Airports Projects Investment (Ten Thousand ¥)	Other Utilities Projects Investment (Ten Thousand ¥)	Annual New Total Investment * (Ten Thousand ¥)	Annual Newly Total Projects Quantities
2011	7330	2439	0	9769	13
2012	22,259	1017	0	23,276	55
2013	20,148	6151	3374	29,673	56

Source: Research Center for Environment and Sustainable Development of China Civil Aviation. * The total investment includes the capital funded by CAAC and the capital invested by every ECER projects implementation subject.

**Table 3:** The output appreciation generated by annual new ECER projects of the Air Transportation Industry from 2011 to 2013.

Items (units: 10,000 ¥)	2011	2012	2013
The operating surplus	6849.22	28,123.84	44,962.85
The net taxes on production	108.33	366.96	525.85
The depreciation of fixed assets	687.60	1873.90	1777.23
The compensation of employees	0.00	0.00	0.00
Total value of annual additional projects	7645.14	30,364.70	47,265.94
**Annual accumulated value**	**7645.14**	**38,009.84**	**85,275.78**

**Table 4:** Top 10 Complete consumption coefficients and the complete partition coefficient of 139 sectors of BTH.

Sector	Complete Consumption Coefficient	Sector	Complete Partition Coefficient
Manufacture of Refined Petroleum Products, Processing of Nuclear Fuel	0.40334971	Air Transport	0.196945669
Extraction of Crude Petroleum and Natural Gas	0.29464438	Public Management and Social Organization	0.115877432
Air Transport	0.19671518	Cargo Handling, Transport Agency	0.107556231
Production and Supply of Electricity and Steam	0.09580717	Professional Technique Services	0.091913374
Wholesale and Retail Trade	0.06862760	Processing of Steel Rolling Processing	0.082559448
Monetary Intermediation and Other Financial Services	0.05662297	Transport Via Road	0.067977225
Manufacture of Other Transport Equipment	0.05083344	Wholesale and Retail Trade	0.067848259
Processing of Steel Rolling Processing	0.04649365	Monetary Intermediation and Other Financial Services	0.066388062
Cargo Handling, Transport Agency	0.04237604	Manufacture of Communication Equipment	0.059473182
Mining and Washing of Coal	0.04026107	Business Services	0.057342530

**Table 5:** Marginal consumption propensity and consumption multiplier of BTH from 2011 to 2013.

Year	Marginal Consumption Propensity	Consumption Multiplier
2011	0.356496	0.5540
2012	0.362868	0.5695
2013	0.375767	0.6020

**Table 6:** The economic impact of ECER projects of by the ATI and comprehensive income of ECER projects of the ATI in BTH from 2011 to 2013 (ten thousand ¥).

Years	2011	2012	2013
**The direct income**	2460.23	12,231.67	27,441.98
**BSE**	5187.33	25,790.18	57,860.74
**FSE**	5289.02	26,295.74	58,994.99
**CME**	7166.74	36,630.97	86,862.21
**EIEA**	20,103.32	100,948.56	231,159.92
**The annual accumulated operating surplus**	6849.22	34,973.06	79,935.91
**The annual comprehensive income**	26,952.54	135,921.62	311,095.83

## Appendix A

**Table A1:** Value added coefficient, complete consumption coefficient and complete partition coefficient of 139 sectors of BTH.

Sector	Value Added Coefficient	Complete Consumption Coefficient	Complete Partition Coefficient	Sector	Value Added Coefficient	Complete Consumption Coefficient	Complete Partition Coefficient
Farming	0.65809490	0.00948396	0.00827421	Manufacture of Machinery for Mining, Metallurgy and Construction	0.33121757	0.00793551	0.00658125
Forestry	0.66232362	0.00160568	0.00036001	Manufacture of Machinery for Chemical Industry, Timber and Nonmetal Processing	0.29064418	0.00164671	0.00173513
Animal Production	0.45784935	0.00414203	0.00567587	Manufacture of Machinery for Agriculture, Forestry, Animal Production and Fishery	0.23530930	0.00011866	0.00166339
Fishery	0.55240879	0.00177300	0.00103603	Manufacture of Other Special Purpose Machinery	0.32346857	0.01044556	0.01138952
Support Services to Farming, Forestry, Animal Production and Fishery	0.42785223	0.00054121	0.00274617	Manufacture of Motor Vehicles, Except Parts and Accessories for Motor Vehicles	0.23432010	0.00503332	0.02322162
Mining and Washing of Coal	0.48668547	0.04026107	0.01592184	Manufacture of Parts and Accessories for Motor Vehicles	0.22017552	0.00688963	0.01486032
Extraction of Crude Petroleum and Natural Gas	0.67784955	0.29464438	0.00417251	Manufacture of Railway Transport Equipment	0.27868824	0.00191602	0.00245919
Mining of Ferrous Metal Ores	0.27078909	0.02206412	0.03042053	Manufacture of Boats and Ships and Floating Devices	0.28240476	0.00066540	0.00066575
Mining of Non-Ferrous Metal Ores	0.48923841	0.00413668	0.00039493	Manufacture of Other Transport Equipment	0.31015770	0.05083344	0.00689735
Mining and Quarrying of Nonmetallic Mineral	0.32712220	0.00334709	0.00181540	Manufacture of Generators and Eclectic Motors	0.19549452	0.00187505	0.00298272
Mining Support Activities and Other Mining and Quarryin n.e.c.	0.24341047	0.00123681	0.00420443	Manufacture of Equipments for Power Transmission and Distribution and Control	0.16727462	0.00782830	0.00931105
Manufacture of Grain Mill Products	0.13832790	0.00111869	0.00121742	Manufacture of Wire, Cable, Optical Cable and Electrical Goods	0.20990082	0.00735762	0.00767773
Manufacture of Prepared Animal Feeds	0.15576646	0.00142819	0.00438317	Manufacture of Batteries	0.18733435	0.00096873	0.00770118
Manufacture of Crude and Refined Oils from Vegetable	0.16310035	0.00106132	0.00370546	Manufacture of Household Appliances	0.20539426	0.00033581	0.00315946
Manufacture of Sugar	0.29309175	0.00061953	0.00008414	Manufacture of Other Electrical Machinery and Equipment	0.30958159	0.00148781	0.00228048
Slaughtering and Processing of Meat	0.18278184	0.00251932	0.00252465	Manufacture of Computer	0.12192421	0.00631335	0.00453914
Processing of Aquatic Products	0.23232448	0.00086776	0.00018777	Manufacture of Communication Equipment	0.12423004	0.00158658	0.05947318
Processing of Other Foods	0.21776476	0.00170671	0.00363674	Manufacture of Broadcasting, Television Equipment of Radar and Related Equipment	0.33123257	0.00054312	0.00111618
Manufacture of Convenience Food Products	0.34504947	0.00063443	0.00256413	Manufacture of Audiovisual Apparatus	0.18389076	0.00145535	0.00323154
Manufacture of Milk and Dairy Products	0.19887917	0.00121440	0.00252478	Manufacture of Electronic Components and Parts	0.20156038	0.01279180	0.04235060
Manufacture of Flavoring and Ferment Products	0.27199137	0.00089935	0.00053458	Manufacture of Other Electronic Equipment	0.28263573	0.00020301	0.01121072
Manufacture of Other Food Products n.e.c.*	0.29356508	0.00365770	0.00766440	Manufacture of Measuring Instruments and Meters	0.30774442	0.00422400	0.00854029
Manufacture of Alcohol and Alcoholic Beverages	0.34193329	0.00422413	0.00188450	Other Manufacture	0.23311783	0.00256814	0.00320148
Manufacture of Soft Drinks and Refined Tea Products	0.27025151	0.00696313	0.00338421	Comprehensive Utilization of Waste Resources	0.42887971	0.00501575	0.00233219
Manufacture of Tobacco Products	0.76213530	0.00008918	0.00040446	Repair of Fabricated Metal Products, Machinery and Equipment	0.40629456	0.00471970	0.00142737
Spinning, Weaving and Finishing of Cotton and Chemical Fibers	0.22435333	0.00357610	0.00916149	Production and Supply of Electricity and Steam	0.19043911	0.09580717	0.02982044
Spinning, Weaving and Finishing of Wool	0.23845787	0.00033331	0.00111817	Production and Distribution of Gas	0.27963437	0.00357168	0.00123536
Spinning, Weaving and inishing of Bast and Silk Fibers	0.25095633	0.00011531	0.00004119	Production and Distribution of Water	0.29976591	0.00282607	0.00150234
Manufacture of Knitted and Crocheted Fabrics and Articles, Except Apparel	0.23668137	0.00354964	0.00051150	Construction of Buildings	0.23044404	0.00455016	0.05605056
Manufacture of Made up Textile Articles, Except Apparel	0.29401192	0.00344994	0.00181027	Civil Engineering	0.18669942	0.00000109	0.01919112
Manufacture of Textile Wearing Apparel	0.31842498	0.00313491	0.00715224	Construction Installation Activities	0.41447198	0.00003788	0.00456523
Manufacture of Leather, Fur, Feather and Its Products	0.27903598	0.00065630	0.00600132	Construction Completion and Finishing, Other Construction Activities	0.31615894	0.01015577	0.00270952
Manufacture of Footwear	0.24615226	0.00002430	0.00048226	Wholesale and Retail Trade	0.73610322	0.06862760	0.06784826
Processing of Timbers and Manufacture of Products of Wood, Bamboo, Rattan, Palm and Straw	0.24427915	0.00326586	0.00147456	Transport Via Railway	0.47313872	0.01173610	0.00603715
Manufacture of Furniture	0.25394624	0.00046908	0.00251287	Transport Via Road	0.46357428	0.02663423	0.06797723
Manufacture of Paper and Paper Products	0.22719333	0.00856405	0.00622567	Water Transport	0.19422865	0.01491260	0.03464911
Printing and Reproduction of Recording Media	0.32160578	0.00447675	0.00289300	Air Transport	0.32180275	0.19671518	0.19694567
Manufacture of Stationeries, Musical Instruments, Products of Arts and Crafts, Sports Goods, Games and Toys	0.31053965	0.00213193	0.00276225	Transport Via Pipeline	0.59513156	0.01570324	0.00086432
Manufacture of Refined Petroleum Products, Processing of Nuclear Fuel	0.12580389	0.40334971	0.01314310	Cargo Handling, Transport Agency	0.23809387	0.04237604	0.10755623
Manufacture of Coke Products	0.15422770	0.00556278	0.00822360	Storage	0.22669326	0.00112504	0.01538479
Manufacture of Basic Chemicals	0.21514787	0.01209001	0.01071587	Post	0.47298839	0.00056459	0.02399714
Manufacture of Fertilizers	0.20867100	0.00143761	0.00170702	Accommodation	0.38835318	0.00676270	0.00761059
Manufacture of Pesticides	0.12732769	0.00041968	0.00137720	Food and Beverage Services	0.39619440	0.02105532	0.01085963
Manufacture of Paints, Printing Inks, Pigments and Similar Products	0.24636065	0.00294550	0.00401435	Telecommunication and Other Information Transmission Services	0.53681145	0.01261370	0.02396582
Manufacture of Synthetic Materials	0.28015570	0.00618445	0.00588272	Software and Information Technology Services	0.31948691	0.00165269	0.04957960
Manufacture of Special Chemical Products	0.22699814	0.01156736	0.00535333	Monetary Intermediation and Other Financial Services	0.67209964	0.05662297	0.06638806
Manufacture of Daily-use Chemical Products	0.30941548	0.00128641	0.00187901	Capital Market Services	0.76535576	0.00895409	0.00041444
Manufacture of Pharmaceutical Products	0.27082951	0.00064797	0.02104495	Insurance	0.39547371	0.00632375	0.01038181
Manufacture of Chemical Fibers	0.18204210	0.00102124	0.00125299	Real Estate	0.71790970	0.02130404	0.03462631
Manufacture of Rubber Products	0.25239312	0.00363817	0.00531817	Renting and Leasing	0.56215994	0.00748130	0.00341211
Manufacture of Plastic Products	0.29046660	0.00908359	0.00917636	Business Services	0.36904634	0.02502596	0.05734253
Manufacture of Cement, Lime and Plaster	0.27823446	0.00157101	0.00422344	Research and Experimental Development	0.35856302	0.00128940	0.02532175
Manufacture of Products of Plaster and Cement and Similar Products	0.22342353	0.00100909	0.00364078	Professional Technique Services	0.35692944	0.00481100	0.09191337
Manufacture of Brick, Stone and Other Building Materials	0.23839244	0.00307113	0.00251076	Technique Promotion and Application Services	0.30464171	0.00549408	0.01778830
Manufacture of Glass and Glass Products	0.24724381	0.00261151	0.00306518	Management of Water Conservancy	0.70200573	0.00013141	0.00102277
Manufacture of Cematic and Porcelain Products	0.36553512	0.00030761	0.00056562	Ecological Protection and Environmental Control	0.54614595	0.00079457	0.00157587
Manufacture of Refractory Products	0.23742876	0.00029001	0.00139037	Management of Public Facilities	0.40298102	0.00026765	0.00280499
Manufacture of Products of Graphite and Other Nonmetallic Minerals	0.28145882	0.00088691	0.00150467	Services to Households	0.51721516	0.00059880	0.01123691
Manufacture and Casting of Basic Iron and Steel	0.20831107	0.01757995	0.02450266	Repair of Motor Vehicles, Electronic Products and ouseholds Goods and Other Services	0.37892044	0.01380824	0.02343402
Processing of Steel Rolling Processing	0.18639454	0.04649365	0.08255945	Education	0.58973109	0.00759619	0.04671477
Manufacture of Ferroalloy	0.15375489	0.00139675	0.00040777	HealthCare	0.41398704	0.00012923	0.01972915
Manufacture and asting of Non-Ferrous Metals and Related Alloys	0.22072408	0.01302412	0.00274603	Social Work Activities	0.73139023	0.00007183	0.00035766
Processing of Non-Ferrous Metals Rolling	0.18359761	0.01005773	0.00587403	Journalism and Publishing	0.39946103	0.00103748	0.00675849
Manufacture of Fabricated Metal Products, Except Machinery and Equipment	0.21077530	0.02066896	0.02476300	Radio, Televisions, Movies and Audio-Video Recording Activities	0.40828552	0.00150128	0.01213762
Manufacture of Boiler and Prime Mover	0.21864505	0.00191237	0.00247806	Cultural, Art and Entertainment Activities	0.60462902	0.00013894	0.00271520
Manufacture of Metalworking Machinery	0.21693402	0.00104880	0.00259596	Sports Activities	0.47942641	0.00001831	0.00415866
Manufacture of Lifting and Hand ling Equipment	0.27426288	0.00278585	0.00472780	Amusement and Recreation Activities	0.48539412	0.00078213	0.00131621
Manufacture of Pump, Valve, Compressor and Similar Machinery	0.26708758	0.00173390	0.00732001	Social Security	0.74156563	0.00000286	0.00030117
Manufacture of Movie, office Machinery and Equipment, of Projector and Camera	0.14748173	0.00068815	0.00247928	Public Management and Social Organization	0.55795782	0.00195229	0.11587743
Manufacture of Other General-Purpose Machinery	0.24428034	0.01351929	0.01113402	——	——	——	——
